# Strikingly different effects of cholesterol and 7-ketocholesterol on lipid bilayer-mediated aggregation of amyloid beta (1-42)

**DOI:** 10.1016/j.bbrep.2018.04.007

**Published:** 2018-04-26

**Authors:** Huong T.T. Phan, Naofumi Shimokawa, Neha Sharma, Masahiro Takagi, Mun'delanji C. Vestergaard

**Affiliations:** a School of Materials Science, Japan Advanced Institute of Science and Technology (JAIST), 1-1 Asahidai, Nomi, Ishikawa 923-1292, Japan; b Hanoi National University of Education, 136 Xuanthuy, Caugiay, Hanoi, Vietnam; c Department of Food Science and Biotechnology, Kagoshima University, 1-21-24 Korimoto, Kagoshima City 890-0065, Japan

**Keywords:** Cholesterol, 7-ketocholesterol, Amyloid beta aggregation, Membranes, Lipid vesicles

## Abstract

Oxidized cholesterol has been widely reported to contribute to the pathogenesis of Alzheimer's disease (AD). However, the mechanism by which they affect the disease is not fully understood. Herein, we aimed to investigate the effect of 7-ketocholesterol (7keto) on membrane-mediated aggregation of amyloid beta (Aβ-42), one of the critical pathogenic events in AD. We have shown that when cholesterol is present in lipid vesicles, kinetics of Aβ nuclei formation is moderately hindered while that of fibril growth was considerably accelerated. The partial substitution of cholesterol with 7keto slightly enhanced the formation of Aβ-42 nuclei and remarkably decreased fibril elongation, thus maintaining the peptide in protofibrillar aggregates, which are reportedly the most toxic species. These findings add in understanding of how cholesterol and its oxidation can affect Aβ-induced cytotoxicity.

## Highlights

• Cholesterol and 7-ketocholesterol membranes had different effects on Aβ aggregation.
• Cholesterol-containing membranes considerably accelerated fibril elongation of Aβ-42.
• 7-ketocholesterol membranes remarkably decreased Aβ-42 fibril elongation.

## 1 Introduction

The aggregation of soluble monomeric amyloid beta (Aβ) peptide into fibrils is one of principal pathogenic events in the pathogenesis of Alzheimer's disease (AD), the most common neurodegenerative illness of late life [1]. According to the prevalent model, Aβ aggregation is a nucleation-dependent polymerization process, including two main steps: (i) nucleus formation and (ii) fibril elongation [2]. In the first step, soluble monomeric Aβ, which is generated from the amyloidogenic cleavage of a transmembrane amyloid precursor protein, undergoes a misfolding from random coil to β-sheet structure [3] and/or adopts a conformational switching from mainly α-helical conformation to β-sheet-enriched conformation under various conditions [4]. The β-sheet conformation is prone to self-aggregation, producing dimers, trimmers, and eventually nuclei (n-mers) [2]. In the next step, the formed Aβ nuclei trigger the formation of intermediate oligomers or protofibrils [5], and finally insoluble fibrils. The fibrils, together with other biomolecules, form extracellular neuritic plaques that are recognized as hallmarks of AD [1]. Cell membranes have been reported to serve as an aggregation matrix for Aβ seeding and for facilitating fibrillar Aβ formation [6]. Accumulating evidence shows that Aβ toxicity significantly depends on the aggregated state. Intermediate species including oligomers and protofibrils are reportedly more toxic than soluble monomers and mature fibrils [7], [8]. Therefore, controlling the Aβ aggregation and the formation of neurotoxic species has become one of the emerging therapeutic strategies in the treatment of AD [9], [10].

It has been reported that the oxidation of cholesterol, a prominent structural component and property modulator of membranes [11], accelerates the development of AD [12]. Cholesterol is susceptible to cellular oxidation induced by enzymes or reactive oxygen species (ROS), generating various oxidized derivatives including 24(S)-hydroxycholesterol (24(S)OH), 25-hydroxycholesterol (25OH) 7-ketochoelsterol (7keto), and 7α/β- hydroxycholesterol (7α/βOH). Possessing one or more supplementary oxygen groups such as hydroxyl, carbonyl, and epoxide, these compounds are more hydrophilic than cholesterol. They also differ from cholesterol in three-dimensional shape as well as orientation in membranes [13]. Increasing evidence is now pointing that oxidized cholesterols play important role in facilitating Aβ generation and accumulation [14], [15], Aβ/membrane interaction [16], [17], [18], and neuron death [19]. However, there is little evidence to date on their effects on membrane-mediated aggregation of Aβ. In this study, we aimed to investigate the impact of 7-ketocholesterol (7keto)-containing model membranes on Aβ− 42 aggregation. 7-keto is a major product of reactive oxygen species (ROS)-caused oxidation of cholesterol [13]. The presence of this sterol in membrane changes membrane physical properties such as fluidity [20], [21], thus altering the interaction of membrane with proteins [16]. We have recently reported the high ability of 7keto to facilitate Aβ insertion into lipid bilayers [17], [18]. Since membrane-mediated aggregation of Aβ-42 is dependent on membrane lipid composition including cholesterol [22], [23], [24], we hypothesized that the partial substitution of cholesterol with this oxysterol in membrane is able to affect Aβ aggregation, thereby leading to the formation of neurotoxic species and triggering Aβ toxicity. Unravelling how 7keto influences Aβ aggregation on lipid bilayer is important to understand the impact of cholesterol oxidation in Aβ-induced toxicity.

## 2 Materials and methods

### 2.1 Materials

1,2-Dioleoyl-*sn*-glycero-3-phosphocholine (DOPC) and cholesterol were purchased from Avanti Polar Lipids (USA). 7-ketocholesterol (7keto) was from Sigma-Aldrich (USA). Amyloid beta protein (Human, 1–42) (Aβ-42) and Hilyte Fluor™ 488-labelled (λex=503 nm, λex=528 nm) Aβ-42 were obtained from Peptide Institute Inc. (Japan) and Anaspec, Inc. (USA), respectively. Thioflavin T (ThT), chloroform, Tris(hydroxymethyl)aminomethane (Tris), and methanol were purchased from Tokyo Chemical industry co., Takara Bio Inc., Kanto-Chemical, and Nacalai Tesque (Japan), respectively. Deionized water was obtained using an ultraviolet water purification system (Millipore S.A.S, France).

### 2.2 Lipid vesicle preparation

DOPC (DOPC only), Chol (DOPC/cholesterol = 50/50 M ratio), and 7keto (DOPC/cholesterol/7keto = 50/40/10 M ratio) lipid vesicles were prepared following natural swelling method [25]. Lipid mixture was dissolved in chloroform/methanol (2/1, v/v) in a glass tube at the final concentration of 0.2 mM. The solvent was subsequently removed by evaporating the tube under a gentle nitrogen stream and drying in a desiccator for 3 h, resulting in a thin lipid film at the bottom of tube. The film was swollen with Tris buffer (20 mM, pH = 7.4) overnight at 37 ᵒC to form lipid vesicles. A phase-contrast microscopy (Olympus BX50, Japan) was employed to estimate the vesicle formation.

### 2.3 Aβ-42 incubation

Aβ-42 was incubated following the method as reported previously [26]. First, 200 µM Aβ-42 solutions were prepared by dissolving Aβ-42 powder in 0.02% ammonia and stored at − 80 °C. The peptide solution was then diluted and incubated in the absence or presence of lipid vesicles (lipid vesicles/peptide = 5/4, v/v) in Tris buffer (20 mM, pH 7.4) at 80 µM concentration in various incubation periods (0 h, 6 h, 12 h, 24 h, 36 h, and 48 h). This concentration has been shown to provide quantitative data as well as characterize molecular events [27]. Since aggregation kinetics of the peptide is known to be influenced by various factors including concentration [28], we have maintained the parameters in subsequent experiments. This enables us to compare our findings, and also build up new understandings.

### 2.4 Measurement of Aβ-42 aggregation

The aggregation of Aβ-42 was assessed by ThT fluorescence assay [26]. The peptide incubated in different conditions was diluted in 20 mM Tris buffer at 20 μM concentration and subsequently added into 5 μM ThT solution contained in a transparent cell. The cell was immediately placed in FP-6500 spectrofluophotometer (Jasco, Japan) to detect ThT fluorescence after an excitation at 450 nm and an emission at 483 nm.

### 2.5 Kinetic analysis of Aβ-42 aggregation

The kinetics of Aβ-42-42 aggregation was analysed using the autocatalytic reaction model reported by Sabaté et al. [2]. ThT fluorescence intensity data was fitted to this model using equation *f* = *ρ*{exp[(1 + *ρ*)*kt*]− 1}/{1 + *ρ*exp[(1 + *ρ*)*kt*]} where *f* is the fraction of fibrillar form$\mathrm{; k}=k_{e}a,k_{e}$ is elongation rate constant, a is the initial concentration of Aβ-42 in the solution; $\rho =k_{n}/k$, $k_{n}$ is nucleation rate constant.

### 2.6 Characterization of Aβ-42 aggregate morphology

Atomic force microscopy (AFM) was used to image and characterize the morphology of Aβ− 42 aggregates derived from the incubation of Aβ-42 alone or with lipid vesicles. In order to prepare AFM samples, 5 μM of Aβ-42 solution was uniformly spread and immobilized in a mica plate (Furuuchi Chemical Co., Shinagawa, Tokyo, Japan). Then, the mica was washed three times with 50 μl of deionized water and was dried under the vacuum condition. The sample was measured by AFM (SPA400-SPI 3800, Seiko Instruments Inc., Japan) equipped with a calibrated 20 µm xy-scan, 10 µm z-scan range PZT-scanner and a scanning silicon nitride tip (SI-DF3, spring constant = 1.6 N/m, frequency resonance = 28 kHz, Seiko Instruments Inc.) in a dynamic force mode (DFM). All AFM operations were performed in an automated moisture control box with 30–40% humidity at room temperature. The length and height of Aβ-42 aggregates were analysed using Image J and SPI software, respectively [18].

## 3 Results

### 3.1 Effect of cholesterol- and 7keto-containing model membranes on the kinetics of Aβ-42 aggregation

**Fig. 1:**
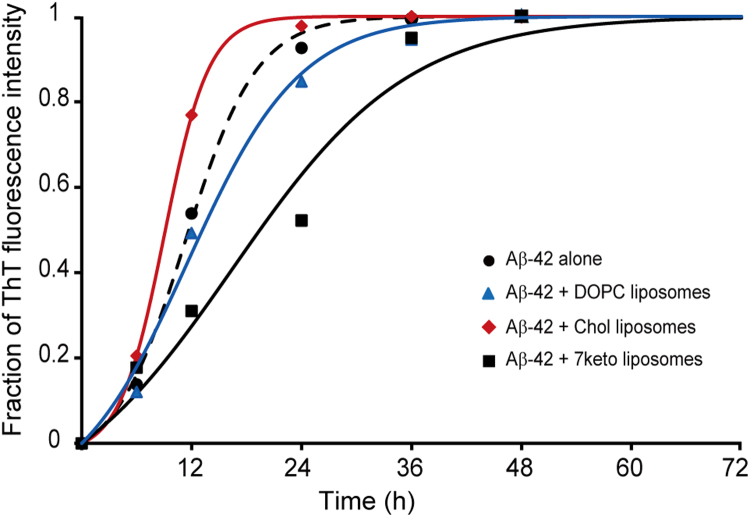
Time course curves of Aβ-42 aggregation in the absence (black, dash) and presence of DOPC vesicles (blue), DOPC/Chol (50/50) vesicles (red), and DOPC/Chol/7keto (50/40/10) vesicles (black, solid). Each point is the average value of three independent experimental measurements. Lines are obtained from fitting the experimental data to the equation of the autocatalytic reaction model [3]. Aβ-42 was incubated at 80 µM in Tris buffer (20 mM, pH 7.4).

First, Thioflavin T (ThT) assay was employed to investigate the effect of cholesterol-containing and 7keto-containing lipid vesicles on Aβ-42 aggregation kinetics in comparison with Aβ-42 aggregation in buffer solution. This assay is a common analytical method for detecting the degree of amyloid fibrillation. Its principle is based on the ability of ThT to show the enhanced fluorescence emission at 483 nm wavelength upon the binding to the β-sheet of Aβ-42 peptide, while that of free ThT is observed at 445 nm [26]. We correlated the fluorescence intensity at 483 nm with the extent of fibrils in solution, as a function of time. Fig. 1 shows time course curves of fibrillar Aβ-42 formation from monomers in the absence and presence of three lipid vesicle systems. The peptide in the absence of lipid vesicles (also in buffer) exhibited a typical sigmoidal curve as reported previously [2], [29]. The sigmoidal curve starts with lag phase in which nucleus formation is detectable, subsequently proceeds on an explosive elongation phase corresponding to a rapid fibril growth, and reaches the equilibrium when most peptide in solution has aggregated into fibrils.

The presence of lipid vesicles changed the time course curve of amyloid fibrillation (Fig. 1). When Aβ-42 was incubated with DOPC vesicles, the lag phase of the curve was slightly shortened, while the elongation phase was significantly delayed compared to the time course curve observed in the absence of lipid vesicles. Conversely, the curve obtained when the peptide was incubated with Chol vesicles was not changed in lag phase, and it had a faster elongation phase. Interestingly, when incubated in the presence of 7keto vesicles, the aggregation kinetics of the peptide was different. The lag phase could not clearly be distinguished from the elongation phase, in contrast to a visible lag phase of time course curves assessed in three other conditions. In addition, the elongation phase was relatively slow. The data suggested that all three studied model membranes influenced the aggregation process of Aβ-42. DOPC and 7keto lipid vesicles decreased the aggregation process, while cholesterol-containing membranes facilitated Aβ-42 fibrillation. The inhibitory effect of 7keto vesicles was higher than that of DOPC systems.

Next, the data of ThT assay was analysed using the Autocatalytic Reaction Model to understand better how lipid vesicles influence the kinetics of two major steps in Aβ-42 fibrillation process, which are nucleus formation and fibril elongation. This model assumes that Aβ-42 fibrillation follows two reactions: (1) $\mathrm{nM}\overset{k_{n}}{\to }P_{n}$ (nucleus formation step) and (2) $M+P_{n}\overset{k_{e}}{\to }P_{n+1\,}$ (elongation step), where M is monomeric peptide, $P_{n}$ is nucleus of fibrils, $P_{n+1}$ is elongated fibril with n+1 molecules of Aβ-42, $k_{n}$ is nucleation rate constant, and $k_{e}$ is elongation rate constant. $k_{n}$ and $k_{e}$ are two key parameters that control the kinetics of amyloid fibrillation process [2]. By fitting the experimental data of ThT intensity to the model using equation *f* = *ρ*{exp[(1 + *ρ*)*kt*]− 1}/{1 + *ρ*exp[(1 + *ρ*)*kt*]} where *f* is the fraction of fibrillar form; $k=k_{e}a$ , a is the initial concentration of Aβ-42 in the solution; $\rho =k_{n}/k$, we assessed the time course curves and kinetics constants of Aβ-42 aggregation. We estimated nucleation rate constant, $k_{n}$, and elongation rate constant, $k_{e}$. Sabaté et al. proposed that $k_{n}\ll 1$ because nucleus formation associates with a series of thermodynamically unfavourable steps, and $k_{e}\gg 1$ since further addition of soluble peptide to nuclei is thermodynamically favourable [2].

As can be seen in Table 1, the assembly of Aβ-42 alone (the control) had a nucleation constant of 3.73 × 10^−6^ s^−1^ and an elongation constant of 3.38 L mol ^−1^ s^−1^. The kinetic constants were considerably influenced by the composition of lipid vesicles (Table 1). When DOPC vesicles were present, the aggregation of Aβ-42 peptide afforded a larger $k_{n}$ and a smaller $k_{e}$ compared the process without vesicles. The kinetic constants demonstrated that DOPC vesicles facilitated nucleus formation and hindered the growth of fibrils. In case of Aβ-42 aggregation with Chol vesicles, nucleation rate constant was decreased by 1.35 fold, while elongation rate constant was increased by 1.65 fold relative to the control. In comparison with DOPC vesicles, this Aβ-42 aggregation had a 2.18-fold lower nucleation rate constant and a 2.83-fold higher elongation rate constant (Table 1). These changes indicated a decreased nucleus formation and a significantly accelerated fibril elongation mediated by cholesterol-containing membranes. Interestingly, the effect of 7keto vesicles on the two kinetic constants was opposite to that of Chol vesicles. In the presence of 7keto vesicles, nucleation rate constant was increased by 1.2 fold, while elongation rate constant was decreased by 2.75 fold compared to control. With respect to Chol vesicles, 7keto systems mediated Aβ-42 fibrillation with a 1.61-fold higher nucleation rate and a 4.54-fold smaller elongation rate (Table 1). These results suggest that 7keto vesicles could maintain the existence of Aβ-42 in intermediate states (oligomers and protofibrils) by accelerating nucleus formation and hindering fibril growth.

**Table 1:** Nucleation and elongation rate constants of Aβ-42 aggregation in the absence and presence of DOPC vesicles (DOPC = 100), Chol vesicles (DOPC/Chol = 50/50), and 7keto vesicles (DOPC/Chol/7keto = 50/40/10). Aβ-42 was incubated at 80 µM in Tris buffer (20 mM, pH 7.4).

Sample	$k_{n}$ (s^−1^)	$k_{e}$ (L mol^−1^ s^−1^)
Aβ-42 alone	3.73 × 10^−6^	3.378
Aβ-42 + DOPC vesicles	5.97 × 10^−6^	1.968
Aβ-42 + Chol vesicles	2.74 × 10^−6^	5.573
Aβ-42 + 7keto vesicles	4.4 × 10^−6^	1.227

### 3.2 Morphology of Aβ-42 aggregates under the influence of cholesterol- and 7keto-containing model membranes

**Fig. 2:**
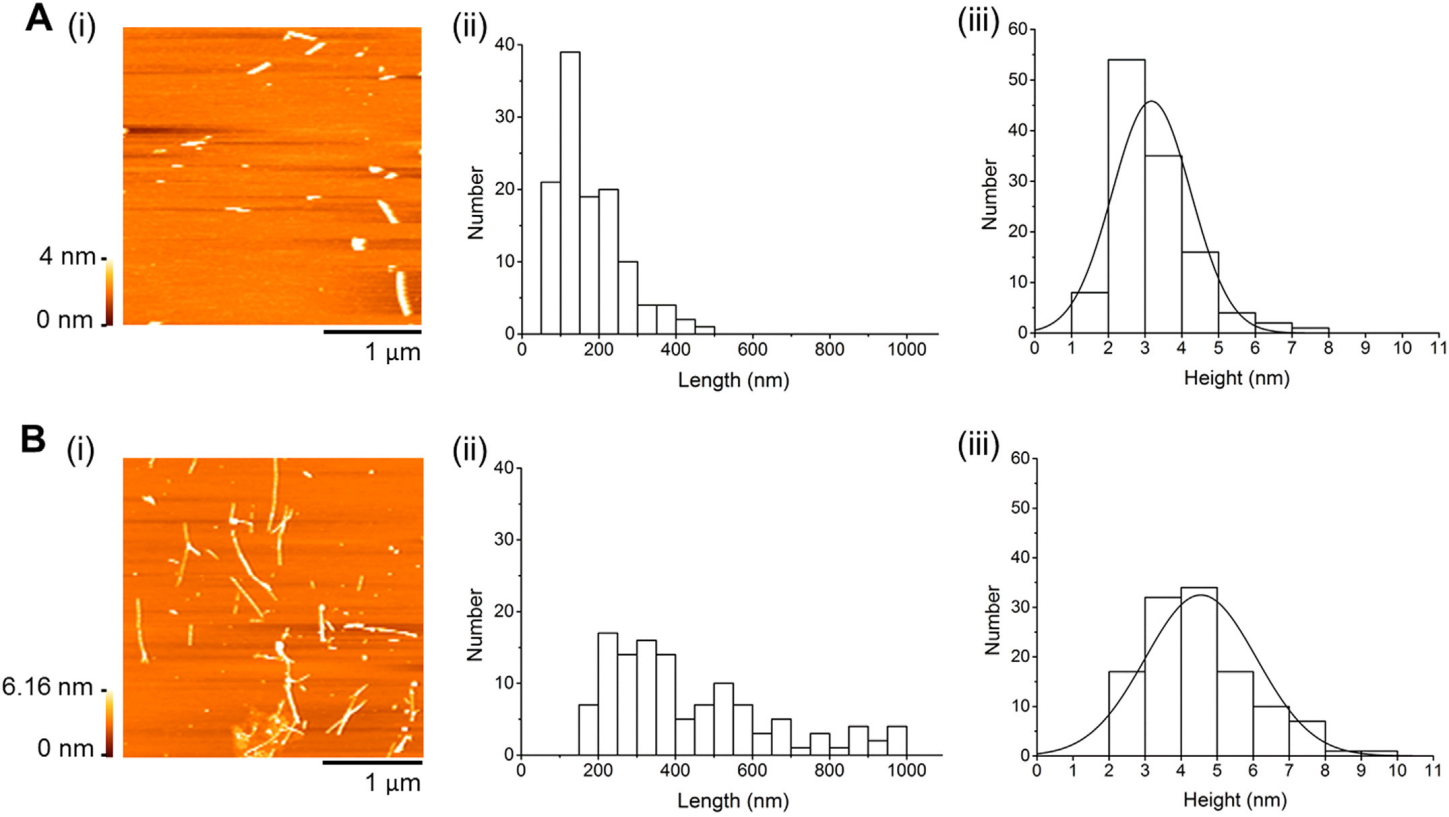
Morphlogy of Aβ-42 aggregates obtained from aggregation in the absence of lipid vesicles. (**A**) Representative AFM images (i), length distribution (ii), and height distribution (iii) of Aβ-42 aggregates obtained from 12-h incubation without lipid vesicles. (**B**) Representative AFM images (i), length distribution (ii), and height distribution (iii) of Aβ-42 aggregates obtained from 24-h incubation without lipid vesicles. Aβ-42 was incubated at 80 µM in Tris buffer (20 mM, pH 7.4).

**Fig. 3:**
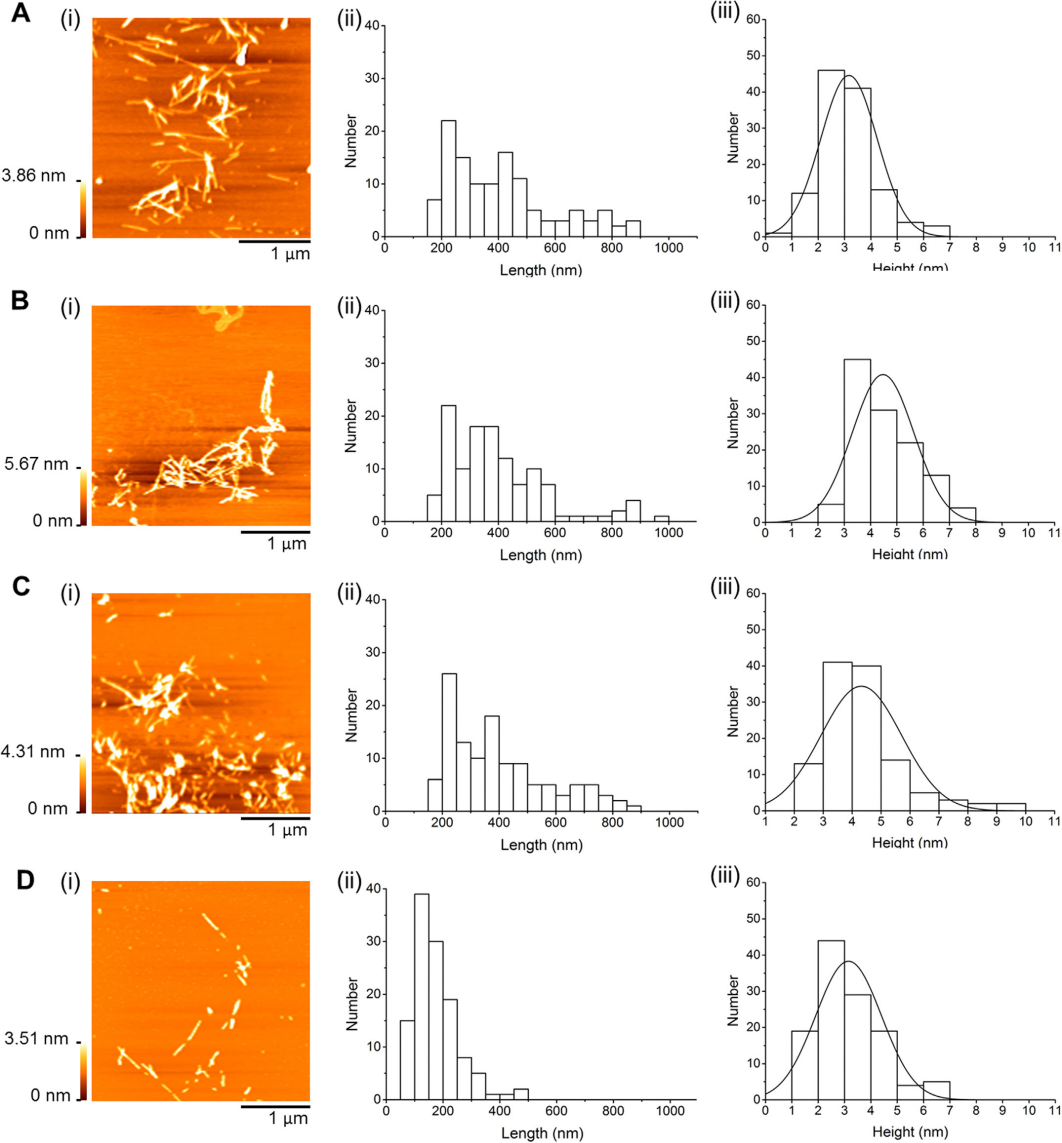
Morphology of Aβ-42 aggregates obtained from aggregation in the presence of lipid vesicles. (**A**) Representative AFM images (i), length distribution (ii), and height distribution (iii) of Aβ-42 aggregates obtained from 24-h incubation with DOPC (DOPC = 100) vesicles. (**B**) Representative AFM images (i), length distribution (ii), and height distribution (iii) of Aβ-42 aggregates obtained from 12-h incubation with Chol (DOPC/Chol = 50/50) vesicles. (**C**) Representative AFM images (i), length distribution (ii), and height distribution (iii) of Aβ-42 aggregates obtained from 24-h incubation with Chol vesicles. (**D**) Representative AFM images (i), length distribution (ii), and height distribution (iii) of Aβ-42 aggregates obtained from 24-h incubation with 7keto (DOPC/Chol/7keto = 50/40/10) vesicles. Aβ-42 was incubated at 80 µM in Tris buffer (20 mM, pH 7.4).

The changes in Aβ-42 aggregation under the effect of DOPC, Chol, and 7keto lipid vesicles were directly visualized using atomic force microscopy (AFM). This imaging technique has been extensively employed in studies of amyloid fibrillation and toxicity due to its ability to capture nanoscale morphological structure of the peptide [30]. We incubated Aβ-42 monomeric solution in the absence and presence of three lipid vesicle systems and characterized the morphology of resultant aggregates. AFM images revealed that after 12 h of incubation alone, peptide aggregates had a rod-like shape with length and height largely in the range from 50 to 250 nm (82.5%) and from 1 to 4 nm (81.1%), respectively (Fig. 2A). This clearly showed that Aβ-42 was mostly protofibrillar [31]. Aβ-42 protofibrils were defined as small elongated oligomers, which are intermediate species in the pathway of fibril formation from soluble monomers [5]. At 24-h incubation, we observed longer, branched or linear aggregates. The aggregates having length of 200–1000 nm and height of 2–6 nn contributed to about 80% of total (Fig. 2B), showing formation of Aβ-42 fibrils [32]. When Aβ-42 was incubated with DOPC vesicles for 24 h, it had branched, fibrillar shape and length distribution similar to the aggregates obtained from 24-h incubation in buffer (Aβ−42 fibrils) (Fig. 3A(i)). However, there was a significant decrease in the height of the aggregates that largely distributed in 1–4 nm range (77%) similar to protofibrils obtained from 12-h incubation of Aβ-42 alone (Fig. 2A(iii) and 3 A(iii)). This result indicates that the formation of fibrillary aggregates was decreased by DOPC vesicles. In the presence of Chol vesicles, amyloid fibrils with typical height and length distributions was observed after only 12 h of incubation (Fig. 3B), implying a relatively faster formation of fibrils than under the other conditions. However, these fibrils tended to form clusters, different from fibrils formed after 24-h incubation (control)_which were not clustered (Fig. 2B and Fig. 3B). As incubation time increased, there was hardly any change in the morphology of fibrils (Fig. 3C). In comparison, Aβ-42 aggregates formed after 24-h incubation with 7keto-vesicles mainly had protofibrillar species (Fig. 3D). 85% of aggregates were 50–250 nm long and 1–4 nm high. This indicates that Aβ-42 fibril formation from monomers was hindered in presence of 7keto vesicles, contrasting the fibril acceleration we had observed in the presence cholesterol-containing membranes. In agreement with ThT assay data, the morphology of peptide aggregates obtained from AFM experiment clearly showed the different effects of cholesterol- and 7keto-containing vesicles on Aβ-42 aggregation.

## 4 Discussion

Our results have revealed that membrane lipids mediated self-assembly of Aβ-42 and this effect was dependent on lipid composition. It has reported that Aβ-42 can aggregate on membrane surface and this aggregation is more favored than in buffer systems [33]. In agreement, we have shown that the formation of Aβ-42 nuclei was promoted by the addition of DOPC vesicles, indicated by a higher nucleation rate constant compared to aggregation without lipid vesicles [Table 1]. Moreover, DOPC vesicles decreased the elongation of nuclei to form fibrils, consistent with a previous study of Aβ-42 assembly on planar lipid bilayers which reported that a 24-h incubation of the peptide on DOPC bilayers produces a lower fraction of fibrils relative to the incubation in buffer [23]. The mechanism of Aβ-42 aggregation on monosialotetrahexosylganglioside (GM1)-free lipid bilayers was discussed in some previously studies [23], [34]. The authors demonstrated that Aβ-42 monomers interacted with lipid bilayers upon binding of the hydrophilic N (1–27 residues) terminus of the peptide to polar head group of phospholipid. Hydrogen bonds can be formed between hydrophilic residues with phospholipid's carbonyl oxygen and phosphate oxygen groups. In addition, the hydrophobic C (28–42 residues) region of the peptide can position into the nonpolar interior of lipid bilayers by hydrophobic interaction. Aβ-42 subsequently undergoes a transition from α-helix-enriched to β-sheet-enriched structures which initiate the nucleus formation and fibril growth [34]. Because lipid bilayers composed of unsaturated DOPC exists in fluid phase, Aβ-42 may insert deeply into the bilayer, by which the progressive aggregation of the peptide is affected [23].

The effects of cholesterol and 7keto on lipid bilayer-mediated fibrillation of Aβ-42 are interestingly different. The presence of cholesterol in pure DOPC vesicles inhibited the nucleation from monomers, but it considerably accelerated nuclei to form fibrils (Table 1, Fig. 1, Fig. 3). This finding was in agreement with some previous studies suggested that cholesterol promoted the already-formed aggregation of Aβ-42 on 1,2-dipalmitoyl-*sn*-glycero-3-phosphocholine (DPPC)/cholesterol [35] and sphingomyelin (SM)/cholesterol mixed bilayers [34]. The nucleation (in lag phase of Aβ polymerization process) depends on peptide concentration [2]. Our previous study showed that cholesterol can reduce the association of Aβ with model membranes due to its ability to pack membrane phospholipids tightly and render DOPC membranes rigid [17]. In this study, we proposed that the decreased amount of Aβ-42 monomers in association with Chol vesicles can account for an inhibited nucleation. Moreover, a molecular dynamics study pointed out that cholesterol had a higher hydrogen bonding affinity with Aβ than headgroup of phospholipids. This sterol is able to compete with peptide-peptide binding during nucleation by forming hydrogen bonds with the peptide [34]. Therefore, the formation of nucleus was slowed down. However, when nuclei were already formed, its elongation and fibril formation were enhanced. As discussed previously, Chol membranes are more rigid than DOPC membranes, thus Aβ-42 was not able to penetrate deeply into the bilayer of cholesterol-containing membranes, and preferentially adsorbed on the surface. As a result, the rate of fibril elongation may be increased [36]. The impact of cholesterol-containing lipid vesicles on Aβ-42 fibrillation was significantly changed by a partial substitution of cholesterol with 7keto. 7keto-containing membrane slightly increased Aβ-42's nucleation rate and remarkably decreased fibril elongation in membranes (Table 1, Fig. 1, Fig. 3). Despite numerous studies on membrane-mediated Aβ-42 aggregation, this is the first report on how cholesterol oxidized derivatives influence the process. Previously, we demonstrated that 7keto renders lipid bilayer less condensed and more fluid than cholesterol, thus accelerating Aβ-42 association with the bilayer [17]. The higher amount of the peptide in membranes may result in an increased nucleation rate. However, Aβ-42 can insert deeply into the bilayer [16], [17], thus the further elongation of the peptide is hindered.

These findings add in understanding cholesterol's impact on membrane-mediated Aβ-42 aggregation on model membranes. In addition to its previously reported effect on GM1/ Aβ-42 binding which seeds the nucleus of Aβ aggregation [37], cholesterol is able to increase the fibrillation process by influencing the interaction of the peptide with lipid bilayer of membranes. Cholesterol accelerated amyloid nuclei to assemble into fibrils, which had a low ability to localize in cell membranes. This effect is disrupted by its oxidized derivatives, 7keto. The sterol decreased Aβ fibrillation and maintained the peptide in protofibrillar aggregates which are reportedly more able to interact with membranes and more toxic than soluble monomers and mature fibrils. Excess cholesterol has been reported as a risk factor in AD's pathogenesis [38], [39]. We propose that high excess cholesterol levels may be readily oxidized in the presence of ROS, leading to formation of oxidized cholesterol which plays significant role in Aβ-induced toxicity due to its ability to hinder the aggregation from more toxic protofibrils to fibrils.

In conclusion, we have shown that cholesterol and 7keto have strikingly different effects on membrane-mediated aggregation of Aβ-42. The presence of cholesterol in lipid vesicles moderately inhibited the kinetics of nucleus formation and considerably accelerated fibrillar Aβ-42 growth. Partial substitution of membrane cholesterol with 7keto slightly increased the formation of nuclei from monomers and remarkably decreased fibril elongation. The results suggested that cholesterol and 7keto can modulate interaction of Aβ-42 with cell membranes by influencing the fibrillation of the peptide, albeit in contrasting ways.
